# One consensual depression diagnosis tool to serve many countries: a challenge! A RAND/UCLA methodology

**DOI:** 10.1186/s13104-017-3111-x

**Published:** 2018-01-03

**Authors:** P. Nabbe, J. Y. Le Reste, M. Guillou-Landreat, E. Beck-Robert, R. Assenova, D. Lazic, S. Czachowski, S. Stojanović-Špehar, M. Hasanagic, H. Lingner, A. Clavería, M. I. Fernandez San Martin, A. Sowinska, S. Argyriadou, C. Lygidakis, B. Le Floch, C. Doerr, T. Montier, H. Van Marwijk, P. Van Royen

**Affiliations:** 1 EA 7479 SPURBO, Department of General Practice, Université de Bretagne Occidentale, Brest, France; 2 EA 7479 SPURBO, Department of Addictology, Université de Bretagne Occidentale, Brest, France; 3 Department of General Practice, Medical University of Plovdiv, Faculty of Medicine, Plovdiv, Bulgaria; 4 Department of Family Medicine “Andrija Stampar”, School of Public Health, School of Medicine, University of Zagreb, Zagreb, Croatia; 5 Department of Family Doctor, University Nicolaus Copernicus, Torun, Poland; 6 Department of Family Medicine “Andrija Štampar” School of Public Health, University of Zagreb, Zagreb, Croatia; 7 Department of General Practice, University of Sarajevo, Sarajevo, Bosnia and Herzegovina; 8 Allgemein Medizin Hochschule Hannover, Hannover, Germany; 9 Galician Health Services, Instituto de Investigación Sanitaria Galicia Sur, Vigo, Spain; 10 IDIAP Jordi GOL Unitat de Support a la Recerca, Barcelona, Spain; 11 Department of English, Nicolaus Copernicus University, Torun, Poland; 12 The Greek Association of General Practitioners (ELEGEIA), Thessaloniki, Greece; 13 Institute for Health and Behaviour, Research Unit INSIDE, University of Luxembourg, Luxembourg, Luxembourg; 14 Allgemein Medizin Hochschule Göttingen, Göttingen, Germany; 15 Unite INSERM 1078, SFR 148 ScInBioS, Faculté de Médecine, Université de Bretagne Occidentale, Université Européenne de Bretagne, 22 Avenue Camille Desmoulins, 29238 Brest Cedex 2, France; 16 Division of Population Health, Health Services Research and Primary Care, School of Health Sciences, Faculty of Biology, Medicine and Health, The University of Manchester, Williamson Building, Oxford Road, Manchester, M13 9PL UK; 17 Department of Primary and Interdisciplinary Care, Faculty of Medicine and Health Sciences, Universiteit Antwerpen, Antwerp, Belgium

**Keywords:** RAND/UCLA appropriateness method, Multicultural consensus, Delphi procedure, Depression diagnosis tool

## Abstract

**Objective:**

From a systematic literature review (SLR), it became clear that a consensually validated tool was needed by European General Practitioner (GP) researchers in order to allow multi-centred collaborative research, in daily practice, throughout Europe. Which diagnostic tool for depression, validated against psychiatric examination according to the DSM, would GPs select as the best for use in clinical research, taking into account the combination of effectiveness, reliability and ergonomics? A RAND/UCLA, which combines the qualities of the Delphi process and of the nominal group, was used. GP researchers from different European countries were selected. The SLR extracted tools were validated against the DSM. The Youden index was used as an effectiveness criterion and Cronbach’s alpha as a reliability criterion. Ergonomics data were extracted from the literature. Ergonomics were tested face-to-face.

**Results:**

The SLR extracted 7 tools. Two instruments were considered sufficiently effective and reliable for use: the Hospital Anxiety and Depression Scale and the Hopkins Symptoms Checklist-25 (HSCL-25). After testing face-to-face, HSCL-25 was selected. A multicultural consensus on one diagnostic tool for depression was obtained for the HSCL-25. This tool will provide the opportunity to select homogeneous populations for European collaborative research in daily practice.

## Introduction

Primary care is a strategic place for depression diagnosis and treatment [1–5]. This led to a triple challenge:

- Improve early diagnosis.
- Provide a simple and effective diagnostic tool that allows medical research in daily practice.
- Gain consensus on the tool’s use irrespective of nationality.

For medical research, there are common selection criteria: efficiency, reliability and ergonomics. The tool must be consensually accepted by researchers and have face validity. It must be validated to indicate when psychiatric referral is required and should be accepted by both psychiatrists and General Practitioners (GPs) [6, 7]. Under the auspices of the European General Practice Research Network (EGPRN), European GP researchers decided to find such a tool. Experts representing different cultures, languages and health systems sought consensus [6, 8].

Seven tools were found using a systematic literature review. They needed to be validated against a psychiatric examination using the DSM’s major depression criteria, usable in primary care research and conceptually understandable by GPs and psychiatrists [9]. Consequently, this method of selection excluded tools such as PHQ, which are not validated against the DSM [10]. Then it was necessary to select the more reliable, efficient and ergonomic tool.

Based on these criteria, the research question was: which diagnostic tool for depression would GP researchers select as the most efficient, reliable and ergonomic for use in clinical research?

## Main text

### Method**s**

#### Criteria to compare

The psychometric properties, (sensitivity, specificity, positive and negative predictive values) of the tools were extracted [9]. They did not vary sufficiently to allow statistical comparison, as the study populations were different. Subsequently, a narrative review was undertaken to extract the reliability data (Cronbach’s alpha, Cohen’s kappa). The ergonomics were also important, but comparing this aspect of tools was complex due to the number of items, test duration, method of inquiry, score range, etc. A consensus, taking into account quantitative and qualitative criteria, based on an European expert panel, was the only alternative to ensure comparison [11].

#### Consensus procedure

The RAND/UCLA appropriateness method (RAM) is approved by major institutes, such as the NICE (National Institute for health and Clinical Excellence) in the United Kingdom or the HAS (Haute Autorité de Santé) in France. It was the most appropriate consensus method [12, 13].

Developed in the mid-1980s, it is an instrument to enable the measurement of the overuse and underuse of medical and surgical procedures. It allows a consensual choice in the comparison of complex processes [11].

**Fig. 1:**
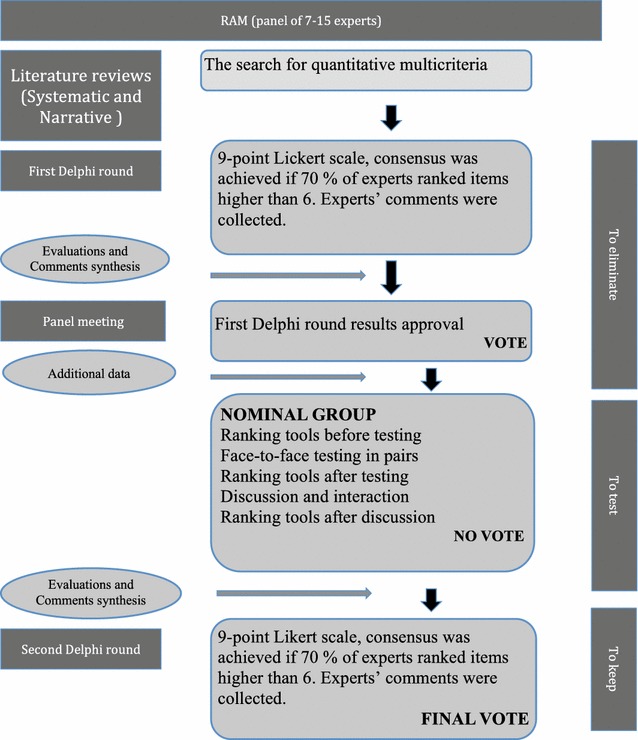
The RAM flow: descriptive diagram of the entire consensus procedure by RAND/UCLA or RAM

RAND/UCLA is a “two-round modified Delphi process” which includes a nominal group. The Delphi rounds avoid leader opinion influence; the panel meeting creates the opportunity to discuss ratings and judgments face to face [14] (Fig 1).

Based on the result of a narrative review completed initially, the quality level of the RAM is increased when the results of a systematic review are used [11, 14].

The RAM is one of several methods that was developed to identify the collective opinion of experts [11]. With RAM, repeated assessment is used by all experts to rank relevance, objectivity and homogeneity [13]. The RAM produces appropriateness criteria and quality indicators with face, construct and predictive validity [15].

#### Experts’ panel

The experts’ panel was purposively selected from primary care, on research expertise, academic expertise, English level, gender, practice, native culture and language [16].

#### First step

The study started with a Delphi procedure to eliminate the less efficient and keep the more reliable tools. The comments took into account only validity data, not ergonomics.

Each expert received the study flow-chart; study method; efficiency, sample and reliability data and consent form. They had to rate the efficiency and reliability of each tool on a 9-point Likert scale [17]:

- Is this tool efficient for the diagnosis of depression in primary care?
- Is this tool reliable for the diagnosis of depression in primary care?

Consensus was defined as at least 70% of the experts rating questions at 7 or above [13]. A tool was considered appropriate if it scored higher than 70% on each question. Comments were collected in order to structure the experts’ panel meeting.

#### Second step

The 2nd step (panel meeting) had to confirm the results of the 1st step and allow debate, without voting, resulting in a presentation of the selected tools. The following resources were provided to experts: methodology reminder, first-round results including all comments, ergonomic features, bibliography data and three 9-point Likert scale notation forms. The forms were completed at the beginning, after testing tools, and at the end of the experts’ meeting.

The experts were invited to discuss the results of the first round and whether they agreed with them. If more than 70% of the experts agreed with the results, the first Delphi round was considered successful.

The experts were invited to rate the following statements:

- “This tool is easy to use in general practice”.
- “This tool could easily be introduced during a consultation”.
- “This tool could be understood by patients”.
- “I like this tool”.
- “Patients could be surprised by this tool”.

Experts were invited to evaluate before and after testing the tools face-to-face in pairs. This was undertaken to assess whether testing tools had modified their judgment. Then the ergonomics were discussed. The meeting ended with final evaluations. The entire meeting was recorded in both video and audio format for ultimate quality control.

No final consensus was required at the end of the meeting [11].

#### Third step

The goal was to select one tool. At the end of the experts’ meeting, all discussions were transcribed. Each expert received the transcript independently.

The final question was: “Which is the most appropriate tool for the diagnosis of depression in adult patients, in General Practice, in Europe, in terms of Efficiency, Reproducibility and Ergonomics?” The experts were asked to vote on each tool and to comment on their responses.

### Results

Eleven experts from 8 European countries participated. They were all GPs, fluent in English. The panel was composed of 9 women and 2 men. Of the 11 experts, 9 practised in urban areas of more than 5000 inhabitants and 2 worked in urban areas with 2000–5000 inhabitants (Table 1).

**Table 1:** Expert panel-participants’ characteristics

Experts	Gender	Country	University statement	Number of inhabitants	Office type	Number of International publications*	Years of practice	Years of research
8	F	Bosnia	Teacher/	2000–5000	GP group office	2	22	12
			Researcher					
10	F	Bulgaria	Teacher/	> 5000	GP group office	9	14	12
			Researcher					
7	F	Croatia	Teacher/	> 5000	Alone	6	20	12
			Researcher					
9	F	Croatia	Teacher/	> 5000	GP group office	18	30	20
			Researcher					
5	F	Germany	Researcher	2000–5000	Stopped practising 2 years earlier	19	23	5
11	F	Germany	Researcher	> 5000	GP group office	4	18	7
3	F	Greece	Teacher/	> 5000	GP and paramedic group office	14	30	18
			Researcher					
4	M	Italy	Researcher	> 5000	GP group office	23	7	6
6	M	Poland	Teacher/	> 5000	GP group office	20	30	12
			Researcher					
2	F	Spain (Cataluña)	Teacher/	> 5000	GP group office	13	22	25
			Researcher					
1	F	Spain (Galicia)	Teacher/	> 5000	GP group office	15	20	14
			Researcher					

* PubMed database

The tools selected by the literature review were: GDS-5, 15 and 30 (Geriatric Depression Scale with 5, 15 and 30 items), the HSCL-25 (Hopkins Symptoms Checklist with 25 items), the HADS (Hospital Anxiety Depression Scale), the PSC-51 (physical symptom checklist in 51 items), and the CES-DR (Center for Epidemiologic Studies Depression Scale-Revised).

#### First step results

The PSC-51, GDS-30 and CES-DR: eliminated for lack of efficiency.

The GDS-15 and GDS-5: eliminated for lack of reliability.

The HADS and the HSCL-25: considered efficient and reliable (Table 2).

**Table 2:** Results of the first Delphi round

	Efficiency		Reliability		Conclusions
	Median (average)	Scores > 6 as percentage	Median (average)	Scores > 6 as percentage	
PSC 51	5 (5)	0	7 (6.9)	80	Eliminated tools: reliable but not efficient
GDS 30	4 (3.6)	0	7 (7.3)	90	
CES DR	4 (3.8)	0	8 (8.1)	90	
GDS 15	8 (7.7)	100	6 (6.6)	0	Eliminated tools: efficient but not reliable
GDS 5	7 (7.4)	91	2 (1.8)	0	
HADS	7 (7.2)	91	7 (7.4)	100	Selected tools: considered both efficient and reliable
HSCL 25	7.5 (7.3)	82	9 (8.5)	100	

#### Second step results

Eight experts participated and confirmed that HSCL-25 and HADS were the best-validated tools in terms of efficiency and reliability.

Before the ergonomics test, the experts had favoured HADS. Their individual opinions were modified after testing the HSCL-25 face-to-face (Table 3). Consensus was not sought at the end of the meeting.

**Table 3:** Evaluation progression during the experts’ meeting

Tools	Statements put to the experts	Scores > 6 as percentage on a 9-point Likert scale		
		First evaluation: after reading only usable data	Second evaluation: after testing and discussion of the questionnaires in pairs	Third evaluation: after discussion among all the experts
HADS	This tool is easy to use in GP’s practice	50	12.5	12.5
	This tool could easily be introduced during a consultation	25	12.5	12.5
	This tool could be understood by patients	37.5	12.5	12.5
	I like this tool	25	12.5	12.5
	Patients could be surprised by this tool	75	62.5	62.5
HSCL-25	This tool is easy to use in GP’s practice	87.5	100	100
	This tool could easily be introduced during a consultation	87.5	75	75
	This tool could be understood by patients	87.5	62.5	75
	I like this tool	87.5	87.5	87.5
	Patients could be surprised by this tool	25	0	0

All comments were collected and were returned to the experts in the document they were sent for the 3rd phase (for example):

*HADS: The questions are difficult for patients to understand; the answers are difficult for patients because they correspond to positive and negative choices; this tool is too long.*

*HSCL*-*25: The answers are on a 1 to 4 Likert scale; the responses are recorded by checking on a table; the answers are simpler.*

#### Third step results

The 8 experts who participated in the whole procedure were asked to vote:

“Which is the most appropriate tool to diagnose depression in adult patients in General Practice, in Europe, in terms of its efficiency, its reliability and its ease of use?”

- 6 answered, “In my opinion, the HSCL-25 is the most appropriate tool to diagnose depression in Primary Care practice.”
- 2 answered, “In my opinion, the HADS is the most appropriate tool to diagnose depression in Primary Care practice.”

The experts gave final comments (for example):

*“After analysing all the psychometric properties, the most useful test in primary care in many countries in Europe, with numerous cultural variations, is the HSCL*-*25.”*

*“In terms of effectiveness, reliability and ergonomics, the HSCL*-*25 is my first choice. However, I must add that the HADS is the best*-*known and most commonly applied tool in clinical practice, as well as in scientific discussions between different medical and non*-*medical professionals. In communication and discussion with our colleagues, it is crucial for the monitoring of depressed patients; we have to think about this if we choose the HSCL*-*25.”*

*“The HSCL*-*25: Simple, detailed enough for the diagnosis, short administration time, easy to understand.”*

### Discussion

The HSCL-25 appeared the most interesting tool for diagnosing depression in terms of the combination of its efficiency, reliability and ergonomics. It is a self-rating scale derived from the SCL-90 which is a multidimensional psychological test instrument for the assessment of psychological symptoms and distress [18–20]. It has robust efficiency and reliability scores [21–23].

This RAM study was based on a systematic literature review [9], of higher quality than the original RAM with a non-systematic literature review. The ergonomic factor was an important criterion in maintaining a relationship between patients and GPs. Researchers demonstrated by this process how ergonomics were decisive in choosing a tool suitable for future research [24].

HSCL 25 has been widely used for evaluation among traumatised populations and used many times in primary care [25–29]. HADS has been widely used over a long period for clinical and research purposes [30]; has been translated into several languages [31] and validated for use in primary care. Nevertheless, HADS seemed complicated for research purposes in daily practice [32–34].

The PSC-51, the CES-DR [35] and the GDS (GDS-30) were considered but efficiency was too low. The GDS was developed specifically to detect depression in elderly patients [36]. It was rejected in the 2 shorter versions: GDS-15 and GDS-5 as reliability was too low [37–41].

In conclusion, the HSCL-25 best combined efficiency, reliability and ergonomics for diagnosis of depression within European primary care practice from a research perspective. It will allow multi-centred collaborative research throughout Europe. HSCL-25 could allow transversal research between psychiatrists and GPs. The group will be vigilant as a self-administered questionnaire must be easily understood by the general population. Its translation into several European languages allows collaborative research. Application in practice must be demonstrated for each national translation.

## Limitations

The quality of the panel was important for the overall quality level. The panel conformed to the requirements of variability in culture, language and practice. 4 language families were represented: Germanic, Slavic, Hellenic and Romance. The panel size was sufficient (7–15 experts) [11].The deadlines for the Delphi rounds were short. Each judgment was performed blind [42]. To reduce information bias, each expert received a record of all the bibliographic sources of the data provided.

The reliability data were mainly based on Cronbach’s alpha values. Those values were extracted using an additional literature review [43].

The tools found in literature were not anonymised. The judgment of each expert could possibly take his/her knowledge into account. Nevertheless, the experts’ opportunity for debate during meetings controlled this possible confusion bias.

A systematic literature review creates the possibility of original selection bias. From the outset, the gold standard was the psychiatric examination based on the DSM’s major depression criteria. Tools with a high level of validity but which did not use this gold standard as their starting point, such as PHQ [44], could not be selected. The objective of the SRL was to focus on the tools; the list was not exhaustive. It could be worthwhile to initiate a study using another gold standard, such as the Hamilton test [45], and compare results.

## Abbreviations

DSM: Diagnostic and Statistical Manual of Mental Disorders
EGPRN: European General Practice Research Network
SRL: systematic review of literature
RAND: Research and Development
RAM: RAND appropriateness method
RAND/UCLA: Research and Development/University of California Los Angeles
NPV: negative predictive value
PPV: positive predictive value
Se: sensitivity
Sp: specificity
